# Reframing Micronutrient Deficiencies for Modern times: A Review

**DOI:** 10.1007/s11606-025-09481-y

**Published:** 2025-03-31

**Authors:** Azadeh Lankarani-Fard, Maria Romanova, Zhaoping Li

**Affiliations:** 1 Department of Medicine Hospitalist Division, VA Greater Los Angeles Healthcare System, UCLA David Geffen School of Medicine, Los Angeles, CA USA; 2 Department of Medicine and Nutrition, VA Greater los Angeles Healthcare System, Chief of Medicine, Chief of the Division of Clinical Nutrition, UCLA David Geffen School of Medicine, Los Angeles, CA USA

**Keywords:** micronutrient deficiency, vitamin deficiency, food insecurity

## Abstract

Micronutrient deficiencies are often discounted in as an entity of the past when access to quality nutrition was scarce. However modern-day conditions such as hemodialysis, complex medication interactions, parenteral nutrition, gastrointestinal resections, institutional living, and substance use can place patients at risk. The metabolic demands of critical illness during prolonged hospitalization may provide added stressors. Food insecurity with reliance on inexpensive calorie-rich, nutrient poor diet may lead to deficiency without overt evidence of malnutrition. Moreover, clinical presentation may be subtle and easily attributed to other diagnoses. Increased awareness of current risk factors is essential for detection and treatment.

## Introduction

Micronutrient deficiencies can be overlooked as they can mimic a multitude of diseases more common in the modern setting. Such presentations can often be confused with familiar diagnoses: “non-ischemic cardiomyopathy,” “vasculitis,” “fungal skin infection,” or “unspecified dementia (Table 1).” Moreover, traditional description of patients with nutritional deficiencies are often framed in the context of gaunt and cachectic individuals, suggesting overall lack of caloric intake. However, several studies and case reports have described patients presenting with vitamin deficiencies despite adequate caloric intake. Modern day conditions such as nutrient-poor-calorie-rich processed foods, complex medication interactions, certain comorbidities, and alcohol or substance use may increase risk for malnutrition.^1,2^ Poor quality nutrition as a result of food insecurity or cognitive impairment requiring institutional living should also be considered.^3^ Reframing the thought process is essential in diagnosing these treatable conditions.

**Table 1:** Common Presentations of Micronutrient Deficiencies

Presentatation	Deficiency
Confusion	• Thiamine—short term memory loss and confabulation • Niacin- confusion is associated with skin findings • Vitamin B12
Ataxia/Peripheral Neuropathy	• Thiamine • Vitamin B12- commonly affects dorsal/lateral columns • Copper
Cardiomyopathy	• Thiamine – high output or dilated cardiomyopathy • Niacin – possible
Dermatitis	• Niacin – rough skin in photosensitive areas (pellagra and Casal’s necklace) • Zinc – mucosal edema especially around mouth and perianal areas with associated fistulization
Anemia	• Vitamin B12- macrocytic anemia • Copper- mimics myelodysplastic syndrome even on bone marrow biopsy • Vitamin C- anemia refractory to iron supplementation • Zinc – mild eosinophilia
Hematoma/Perifollicular Hemmorhages/”Easy Bruising”	• Vitamin C
Glossitis	• Vitamin B12
Hemolysis- non-auto-immune mediated	• Vitamin B12

### Thiamine/B1 Deficiency

#### Presentation and Pathophysiology

Thiamine deficiency is more common than expected. A systematic review of community dwelling adults noted it was the second most common micronutrient deficiency after vitamin D.^4^ Although commonly framed as a presentation of confusion in those with alcoholism, thiamine deficiency can also present as unexplained heart failure, called wet or cardiac beriberi. Dry beriberi refers to the neurologic manifestation of thiamine deficiency which can include Wernicke’s encephalopathy, Korsakoff psychosis, peripheral neuropathy, as well as dysautonomia.

Thiamine is essential in the production of ATP, neurotransmitters, and carbohydrate metabolism. Without thiamine, excess carbohydrates are shunted on to fatty acids and proteins creating advanced glycosylated end-products which can cause cell damage and neurotoxicity.^5^

The vitamin is absorbed in the small intestine via active transport though passive absorption can occur at high concentrations. Small amounts of the water soluble vitamin are stored in the liver, muscle, heart, red blood cell as well as the CNS. Stores can be depleted in about 4–6 weeks but can occur faster under certain conditions such as the high catabolic states of severe illness.^6,7^

#### Risk Factors

While its association with alcohol use is well described, other associated risk factors include: chronic dialysis, sepsis, hyperthyroidism, malignancy, and refeeding syndrome.^7–10^ One study noted 10/30 of the hemodialysis patients who presented with confusion were attributed to low thiamine.^9^ A post hoc analysis of randomized trials that evaluated thiamine administration in patients with septic shock showed benefit and reduced need for renal replacement therapies.^11^ Chronic furosemide use has been implicated in thiamine deficiency in some studies.^12^ Case reports of thiamine deficiency have presented as unexplained heart failure and dementia in those with food insecurity and social isolation as well as those who have undergone gastrointestinal resections.^13,14^

#### Diagnosis and Treatment

Diagnosis should be based on clinical suspicion and response to supplementation. Laboratory confirmation can be helpful. However plasma thiamine as well as whole blood thiamine level can be affected by recent caloric intake therefore most diagnostic labs suggest testing after a fasting period. Other laboratory evaluations such as urine thiamine or thiamine pyrophosphate analysis are cumbersome to perform in the clinical setting.^15^ Lactate levels are occasionally elevated in patients with deficiency.

High dose intravenous thiamine is the preferred route for initial repletion especially in those with prolonged deficiency, impaired GI absorption such as those with chronic alcohol use or those with CNS manifestations such as Wernicke’s encephalopathy. Most regimens use intravenous doses in excess of 200mg given 2–3 times a day. The Royal College of Physicians and several other societies recommend intravenous thiamine of 500mg two or three times a day for 2 days then 250mg intravenous daily for another 2–3 days before converting to oral supplementation of 100-200mg a day.^16,17^ Such doses are much higher than the typical “banana bag” or those found in most over-the-counter multivitamin preparations. A review of inpatient thiamine orders showed that only 10–20% of orders were written at sufficient intravenous doses when treating neuropsychiatric symptoms.^18^ Lower doses of intravenous thiamine have been used to treat the cardiac manifestations of thiamine deficiency.^19^ Maintenance therapy of 50-200mg a day in addition to a multivitamin is suggested after repletion. In general, most case reports note neurologic manifestations can be more refractory to treatment than other complications of the disease.

### Niacin/Vitamin B3

#### Presentation and Pathophysiology

Niacin deficiency or pellagra carries the long-standing mnemonic of the 4D’s: “dementia, dermatitis, diarrhea, and death.” Pellagra, which translates to “rough skin” in Italian, is marked by the photosensitive rash in sun-exposed areas that can appear like raw skin. Around the neckline the rash has been described as Casal’s necklace.

Niacin is an essential component of the cofactors NAD(H) and NADP(H), which are involved in catabolic and anabolic metabolism respectively. The vitamin is stored in the liver, kidneys, and erythrocytes however a significant portion of niacin is generated by the liver via the conversion of tryptophan.^20^

Grains, meats, legumes, seeds, and alkali-treated-corn are a source of niacin. The treatment of whole corn via an alkali solution, a process called nixtamalization, releases the unusable niacytin from hemicellulose, making the vitamin absorbable. Tryptophan from protein can also be converted to niacin, though this process is inefficient and requires adequate stores of vitamin B6 and thiamine.

#### Risk Factors

Outbreaks of pellagra historically occurred in populations who relied on inexpensive diets of untreated corn and had limited access to protein. More recently, pellagra has been diagnosed in patients with cirrhosis, malabsorptive states, and post-bariatric surgery.^20,21^ Food insecurity has also been associated with pellagra especially in those with concomitant substance use disorders.^22,23^ Case reports of pellagra encephalopathy have been mistaken for Wernicke’s encephalopathy in patients presenting with alcohol withdrawal and worsened after thiamine repletion without adequate niacin supplementation.^21,24,25^

Medications that prevent the conversion of tryptophan to niacin can also precipitate pellagra. Such medications include: 5-fluorouracil, pyrazinamide, phenobarbital, and azathioprine.^20^ Prolonged use of isoniazid has also been associated with pellagra as the medication depletes stores of vitamin B6, which is required to convert tryptophan to niacin.^26^ Biochemical niacin deficiency even without clinical manifestations can occur in those with carcinoid syndrome as excess tryptophan is shunted to produce serotonin rather than niacin.^20^ Hartnup’s disease, a rare genetic disorder of tryptophan uptake can also lead to pellagra in the context of insufficient intake of dietary niacin.

#### Diagnosis and Treatment

Diagnosis of niacin deficiency relies on the clinical picture and reversal of symptoms with treatment as laboratory tests can be difficult to interpret or obtain. Plasma niacin test evaluates several metabolites of niacin and is the most readily available but can be unreliable. Low NAD/NADP ratios or a low urine levels of methylated niacin metabolites (N(1)-methyl-nicotinamide and N1-methyl-2-pyridone-5-carboxamide) are more reliable indicators of deficiency but can be difficult to obtain.^27,28^

Repletion of niacin can start at 300mg (range 100–500 mg/d) of immediate release niacinamide in 2–4 divided doses until acute symptoms resolve.^29^ The repletion should be followed by the USDA recommended dose of about 14-16mg/day, supplied by most over-the-counter multivitamin formulations. It is important to note that niacinamide (or nicotinamide) differs from the nicotinic acid used for lipid lowering. The latter was associated with flushing, gastrointestinal distress, myopathy and liver dysfunction when used in high doses over 1000mg/day.^29^

### Vitamin B12/Folate deficiency

#### Presentation and Pathophysiology for Vitamin B12 Deficiency

Vitamin B12 and folate deficiencies are often evaluated together as their roles are intertwined. Vitamin B12, or cobalamin, is found in animal derived foods where it is bound to protein. Very little is found in plant-based foods unless the product is fortified. Gastric acid, pepsin, intrinsic factor, and pancreatic proteases need to be present to aid absorption which occurs in the ileum. Conditions that interfere with B12-intrinsic factor formation, decrease gastric acid, or disrupt absorption at the ileum can result in deficiency. Once absorbed, the active form of the vitamin is bound to transcobalamin II while the inactive form is bound to haptocorrin.^30^

The liver stores approximately 2-3mg of the vitamin which is also reabsorbed via the enterohepatic pathway. Given a typical daily requirement of 2.4mcg, it would take years to deplete stores.^30,31^ The vitamin is essential for DNA synthesis, methylation, production of hematopoietic cell as well as maintenance of the myelin sheath.

Patients often present with glossitis, cognitive changes or a symmetrical neuropathy involving dorsal and lateral columns of the spinal cord causing weakness, ataxia, and paresthesias. Laboratory studies may reveal macrocytosis or hypersegmented neutrophils. In extreme cases, a non-autoimmune hemolytic anemia with associated elevated LDH may be present due to abnormal DNA synthesis.^30^ The absence of an anemia should not exclude the disease if other symptoms are present.

#### Risk Factors for Vitamin B12 Deficiency

Approximately 30% of the elderly could be vitamin B12 deficient.^3^ Common etiologies for B12 deficiency include the autoimmune disease pernicious anemia or sequelae of gastrectomy or bowel resection. Resection of > 20cm of the ileum is associated with deficiency. Patients undergoing these resections are given vitamin B12 supplementation routinely. Those on a strict vegan diet without supplementation may develop vitamin B12 deficiency years after their lifestyle change, thus not attributing any symptoms to changes in their diet.^30^

Other lesser-known risk factors for B12 deficiency include: achlorhydria due to proton pump inhibitors or H2 blockers, gastric atrophy due to aging or chronic alcohol use, pancreatic insufficiency or bacterial overgrowth.^30,32^ Long term metformin use has been associated with B12 deficiency and increased risk of neuropathy.^33^ The drug is suspected to interfere with the calcium mediated uptake of vitamin B12 in the terminal ileum, a process that can be reversed by calcium supplementation.^34^

Nitrous oxide can cause a rapid onset of B12 deficiency as it renders vitamin B12 inactive via the irreversible oxidation of cobalt. The inhaled gas is used as an anesthetic. However gas cannisters of nitrous oxide, called whippets, used in the culinary industry to make foam are increasingly used as recreational drugs. Several case reports have noted acute vitamin B12 deficiency due to recreational nitrous oxide use or prolonged nitrous oxide anesthesia.^35,36^ Without an understanding of the underlying cause, patients presenting with a peripheral neuropathy, falls, or confusion can be attributed to irreversible complications of diabetes, dementia, or drug use.

As an aside, elevated levels of vitamin B12 can be attributed to myeloproliferative disease due to increased production of haptocorrin.^37^

#### Presentation and Pathophysiology for Folate Deficiency

In contrast, folates are found in green leafy vegetables, grains, and eggs. Folate is the naturally occurring compound which can be destroyed by cooking. Folic acid is the stable synthetic form of the vitamin found in supplements and enriched products. Leucovorin or folinic acid, is a reduced form of the vitamin used to increase the cytotoxicity of 5FU and prevent deficiency in patients taking methotrexate. The vitamin is essential in DNA synthesis and by extension, hematopoiesis. Folates are absorbed in the jejunum and recirculated via the enterohepatic circulation.^38^ Isolated folate deficiency is rare and often presents with fatigue and anemia. Neurologic symptoms are not as marked as with vitamin B12 deficiency.

#### Risk Factors for Folate Deficiency

Low folate levels usually occur in the context of a restricted diet, bariatric surgery, or malabsorption. Increased cell turnover such as pregnancy, severe eczema, chronic hemolysis, or exfoliative skin disease can contribute as well.^39^ Folate losses can occur with chronic hemodialysis or external biliary drainage.^31^

#### Diagnosis and Treatment of Vitamin B12 and Folate Deficiency

Evaluation for vitamin B12 and folate deficiency often occurs simultaneously. A complete evaluation for deficiency would evaluate vitamin B12, folate, methylmalonic acid (MMA), and homocysteine levels.^30^ Anti-intrinsic factor and anti-parietal cell antibodies should be considered to evaluate for pernicious anemia. Patients on chronic dialysis should have RBC folate levels evaluated rather than serum folate.^31^ Homocysteine levels are increased in both folate and vitamin B12 deficiencies however MMA is only elevated in B12 deficiency. Of note, although elevated homocysteine is associated with increased thrombosis, folic acid supplementation has not been showed to reduce the risk of such events.^40^

A vitamin B12 value < 200pg/ml is indicative of deficiency. Vitamin B12 levels 200-300pg/ml and folate levels between 2-4ng/mL are considered borderline and may warrant further investigation.^30^ Vitamin B12 levels may be normal in those with nitrous oxide overuse as the underlying etiology is inactivation rather than deficiency.^41,42^ However homocysteine and methylmalonic acid levels will be elevated.

Vitamin B12 repletion should factor the underlying cause and severity of symptoms. Intramuscular route is preferred in patients with malabsorption or severe hematologic or neurologic symptoms. Initial dose can range from 1000mcg of intramuscular cyanocobalamin one to three times a week for 4 weeks or once daily for the first week followed by once a week doses for 4 weeks. Once symptoms improve, patients can be maintained on intramuscular preparation of 1000mcg once a month. Oral supplementation of 1000mcg can be used in those with milder symptoms and an intact gastrointestinal tract. A supratherapeutic oral dose has been used to promote passive absorption, bypassing the active transport mediated by intrinsic factor, in those with malabsorption who are unable to take intramuscular injections.^30^

Folate repletion can start at 1 to 5mg a day until anemia improves followed by maintenance dose of 400mcg if the reason for deficiency persists. Vitamin B12 levels should be closely monitored as prolonged high dose folic acid supplementation may improve anemia but worsen an underlying vitamin B12 deficiency, leading to neurologic damage.^43^

### Vitamin C

#### Presentation and Pathophysiology

Vitamin C (ascorbic acid) deficiency or scurvy has been historically associated with sailors crossing the ocean, their diets lacking the water-soluble vitamin commonly found in citrus, potatoes, tomatoes, and green vegetables. Though the vitamin is stored in the liver and red blood cells and partially resorbed by the kidney, the body can be depleted after 3 months of deficient intake. Prolonged cooking, pasteurization, and high-heat canning can destroy the vitamin.^44^

The vitamin acts as a cofactor (electron donor) for enzymatic reactions involved in the synthesis of collagen, neurotransmitters, and nitric oxide. Its ability to reduce dietary iron, allows for more efficient absorption of iron and reduced risk for anemia.

Vitamin C may also have a role in dampening the over-activation of the inflammatory cascade in processes such as sepsis. However clinical trials that have evaluated addition of vitamin C to sepsis regimes have not shown a clear benefit.^45–48^

Presentation can include hyperkeratosis, petechiae, coiled hairs, hemorrhage around the hair follicles, along with easy bruising and gingivitis. Depression, arthralgias, and vasomotor instability have been noted. Anemia may be present due to impaired absorption of iron.

The clinical manifestations can often mimic conditions associated with drug side effects, chronic liver disease, or malignancy. The petechiae and perifollicular hemorrhages can mimic the palpable purpura of vasculitis and initiate an extensive rheumatologic evaluation.^49^

#### Risk factors

In the modern era, individuals reliant on shelf-stable foods, the so called “bachelor’s diet”, may present with scurvy despite overall adequate caloric intake.^50,51^ The disease has been noted to be more common in populations who live in remote areas and are at risk for food insecurity due to low socio-economic status.^52^ Scurvy has also been associated with chronic substance and tobacco use. Studies have suggested that vitamin C deficiency could be more common than thiamine deficiency in those with alcohol use disorder (~ 80% vs 30%).^53^ Some guidelines proposed vitamin C as well as thiamine repletion for those with chronic alcoholism.^54,55^

Low dose oral supplementation in hemodialysis patients has been associated with decreased epoetin and iron requirements.^56^ Low vitamin C levels have been noted in critically ill inpatients;^57,58^ however this should be interpreted with caution as suppressed levels may be due to intracellular uptake as part of an acute phase response.^59^

#### Diagnosis and Treatment

Plasma vitamin C levels are best evaluated after a fasting period as levels can be falsely normal or elevated after recent intake. One study suggests whole blood vitamin C levels to be preferable over plasma and urge caution in interpreting low results in the absence of clinical findings or when C-reactive protein is greater than > 5 mg/L.^60^ Functional assays such as leukocyte vitamin C are more reflective of body stores but not widely available.

It is important to note that most patients with deficient levels may not have the classic findings of scurvy. Plasma ascorbic acid or vitamin C levels between 11–28 µmol/L are considered marginal and may reflect biochemical deficiency before clinical signs are apparent. Such patients may quickly develop scurvy when subjected to a brief period of dietary changes.^61^ Overt symptoms of scurvy are more likely to be apparent when plasma levels are < 11 µmol/L.

Treatment doses will vary. A general regimen of 200mg intravenous ascorbic acid for 7 days is a preferred starting point.^59^ Doses as high as 2–3 g/day have been suggested depending on comorbidities such as critical illness, renal replacement therapies or malabsorption. Oral doses of 50mg-100mg may be sufficient in milder cases. Oral supplementation higher than 100mg is not absorbed well. High dose supplementation after repletion for prolonged period of time can lead to hyperoxaluria and kidney stones.

### Zinc

#### Pathophysiology and Presentation

Zinc is essential in the structural integrity of essential proteins such as histones, zinc-finger proteins, tight junctions, alkaline phosphatase, and DNA/RNA polymerase. Zinc is absorbed through-out the intestine and competes for the same sites of absorption as copper and iron.

In adults, the most severe manifestation is the characteristic acrodermatitis enteropathica. Though commonly described as an autosomal recessive disease in children caused by impaired zinc transport, an acquired form of acrodermatitis enteropathica has been noted in nutritionally impaired adults.^62,63^ The hyperkeratotic “crusting” lesions commonly involves the eyes, mouth and perianal areas and is associated with significant edema and diarrhea. Patients may have poor wound healing with fistulization as zinc deficiency affects tight junctions.^59^ Mild eosinophilia may be present on blood tests as well as tissue samples.^64^

#### Risk Factors

Absorption may be impaired by pancreatic insufficiency, malabsorptive states, inflammatory bowel disease as well as gastrointestinal resections. Pregnancy, alcoholism, cirrhosis, total parenteral nutrition, and diuretics, have also been associated with deficiency. One study noted approximately 70% of hemodialysis patients were noted to be zinc deficient.^65^

#### Diagnosis and Treatment

Plasma zinc levels may be suppressed in the context of severe inflammation however they are accurate when associated with clinical findings.^59^ Modest supplementation about 30 to 45 mg/day is sufficient and should be given two hours after any iron supplementation. Long term treatment especially with doses > 50mg/d should be closely monitored as it has been associated with low copper status as well as side effects of diarrhea and GI distress.^59^

### Copper

#### Pathophysiology and Presentation

Copper deficiency presents with fragile hair, muscle weakness, edema, hepatosplenomegaly, and osteoporosis. Patients may have myelopathy, neuropathy, and ataxia on exam similar to subacute combined degeneration.^66^ Laboratory studies can reveal a pancytopenia and bone marrow dysplasia that mimics myelodysplastic disease, making it standard to exclude copper deficiency when evaluating a patients with potential myelodysplastic syndrome. An anemia that is refractory or worsens with iron supplementation can occur as iron can prevent copper absorption.

#### Risk Factors

Risk factors for acquired copper deficiency include excess zinc intake, chronic malabsorptive states, dialysis, bariatric surgery, bowel resection, and total parenteral nutrition.^67,68^ Excess zinc intake from denture cream adhesive has been implicated in copper deficiency.^69^

#### Diagnosis and Treatment

Low plasma copper, and ceruloplasmin are indicative of deficiency. Repletion dose is largely based on consensus and depends on the adequacy of absorption from the gastrointestinal tract. Most regimens suggest 2mg of elemental copper a day for 1–3 weeks followed by 2mg of elemental copper if on-going deficiency is expected. Cessation of zinc supplementation is important in obtaining a clinical response.^59,70^

## Conclusion

The risk for micronutrient deficiency persists even in the developed world yet it is often not considered. Small studies and multiple case reports have described its clinical presentation which may have been initially overlooked in favor of more modern clinical diagnoses. There is a need for increased awareness of micronutrient deficiencies especially in special populations. Despite the long-standing description of these conditions, there is also a need for updated testing and treatment guidelines, as current protocols are varied and largely based on consensus. The increased interest in the study of nutrition and food insecurity should include risk for micronutrient deficiency in overall evaluations.
